# Incubator-independent cell-culture perfusion platform for continuous long-term microelectrode array electrophysiology and time-lapse imaging

**DOI:** 10.1098/rsos.150031

**Published:** 2015-06-17

**Authors:** Dirk Saalfrank, Anil Krishna Konduri, Shahrzad Latifi, Rouhollah Habibey, Asiyeh Golabchi, Aurel Vasile Martiniuc, Alois Knoll, Sven Ingebrandt, Axel Blau

**Affiliations:** 1 Department of Neuroscience and Brain Technologies (NBT), Italian Institute of Technology (IIT), via Morego 30, Genoa 16163, Italy; 2 Department of Informatics and Microsystem Technology, University of Applied Sciences Kaiserslautern, Amerikastraße 1, Zweibrücken 66482, Germany; 3 Computer Science Department VI, Technical University Munich (TUM), Boltzmannstraße 3, Garching 85748, Germany

**Keywords:** cell culture, benchtop perfusion, MEA electrophysiology, time-lapse imaging, polydimethylsiloxane, replica casting

## Abstract

Most *in vitro* electrophysiology studies extract information and draw conclusions from representative, temporally limited snapshot experiments. This approach bears the risk of missing decisive moments that may make a difference in our understanding of physiological events. This feasibility study presents a simple benchtop cell-culture perfusion system adapted to commercial microelectrode arrays (MEAs), multichannel electrophysiology equipment and common inverted microscopy stages for simultaneous and uninterrupted extracellular electrophysiology and time-lapse imaging at ambient CO_2_ levels. The concept relies on a transparent, replica-casted polydimethylsiloxane perfusion cap, gravity- or syringe-pump-driven perfusion and preconditioning of pH-buffered serum-free cell-culture medium to ambient CO_2_ levels at physiological temperatures. The low-cost microfluidic *in vitro* enabling platform, which allows us to image cultures immediately after cell plating, is easy to reproduce and is adaptable to the geometries of different cell-culture containers. It permits the continuous and simultaneous multimodal long-term acquisition or manipulation of optical and electrophysiological parameter sets, thereby considerably widening the range of experimental possibilities. Two exemplary proof-of-concept long-term MEA studies on hippocampal networks illustrate system performance. Continuous extracellular recordings over a period of up to 70 days revealed details on both sudden and gradual neural activity changes in maturing cell ensembles with large intra-day fluctuations. Correlated time-lapse imaging unveiled rather static macroscopic network architectures with previously unreported local morphological oscillations on the timescale of minutes.

## 1. Introduction

Unlike cells within living organisms, cells *in vitro* lack a supporting body infrastructure. They are not protected by the immune system or other basic system-wide regulatory mechanisms that control the temperature, pH and the turnover of nutrients, metabolites and signalling factors. Nevertheless, they are considered meaningful model systems for investigating functional physiological subsets or for testing *in vivo*-like constructs [1–5]. However, the cell-culture infrastructure (e.g. incubator, sterile hood) required to keep cultures alive is largely incompatible with most screening tools (e.g. microscopy, electrophysiology) and thus limits experimental possibilities. For this reason, most non-sacrificial cell-culture experiments are performed at ambient conditions. To prevent drifts in temperature, pH and osmolality, they rely on taking exemplary, quasi-static data snapshots over short periods only. This practice biases insights into physiological events and their temporal correlation. Typically, the data are collected at different basal activity levels. These independent and fragmented datasets may furthermore be distorted by both environmental and handling artefacts. For instance, manual medium exchange and variations in cell-culture handling during culture transfer from the humidified CO_2_ incubator to the dry experimental set-up are acting as non-reproducible stimuli that modulate cell physiology. By contrast, an incubator-independent and automated perfusion system would stabilize physiological conditions on the experimental set-up and thus allow for the functional separation of environmental variability from physiological fluctuations. It would also extend the experimental time frame. In the best case, a culture system could reach, if not supersede, the natural lifetime of the donor animal that provided the tissue. Various custom-made [6,7] or commercial microscope incubators have been demonstrated to stabilize the pH, temperature and humidity (for a list see Nikon MicroscopyU). In general, they copy the elements and working principle of an incubator in a miniaturized, platform-adapted implementation. Therefore, they share some of the limitations of classical incubators. For instance, without further shielding the exposed electronic circuits or connectors against humidity, a highly humid atmosphere can lead to corrosion and functional failure of electronic data acquisition components. For imaging, optical windows need to be heated to prevent water condensation along temperature gradients. Furthermore, the majority lack an automated medium perfusion function. This shortcoming is addressed by a plethora of cell-culture perfusion and microchannel devices in different experimental contexts, as recently reviewed in general [8–14] and with a special focus on applications in neuroscience [15–17]. However, the majority still depends on classical incubation schemes to keep the cells viable, thereby limiting the experimental possibilities. To get around above described constraints, our two main goals were the decoupling of cell cultures from standard cell-culture infrastructure accompanied by the automation of cell culturing tasks. To this end, we devised a replica-casted perfusion cap and paired it with a simple multi-purpose benchtop perfusion scheme for timed or very slow-flow operation to reduce manually induced interference artefacts. In combination with a chemically buffered medium and a temperature controller, this platform allows for unsupervised multimodal long-term imaging and extracellular electrophysiology studies at ambient conditions immediately after cell seeding that are unbiased by physical and chemical handling artefacts.

## 2. Material and methods

### 2.1 Polydimethylsiloxane cap moulding master templates

Perfusion cap templates were designed (Alibre Design) to fit the standard outer diameter (OD) of 24 mm glass or polymer rings that are usually found on commercial microelectrode arrays (MEAs). The polymethyl methacrylate moulding template consisted of five slidable parts made for the arbitrary definition of vertical cap geometries (electronic supplementary material, figure S1). These were held in place by M4 polymer screws. Part 1 determined the OD of the polydimethylsiloxane (PDMS) lid; parts 2 and 5 determined its total height. Part 3 defined the inner cap diameter, which equalled the OD of the culturing dish; in this case, this was the OD of the Ø 24 mm glass ring. A smooth groove slightly below its upper edge added a sealing O-ring feature to the PDMS cap. Two holes (Ø 2.2 mm) centred at opposite sides of the upper edge of part 3 (or part 4, not shown) provided relative positioning and temporary fixation of the polytetrafluoroethylene (PTFE) inlet and outlet tubes. The shape of the inner cylinder (part 4) defined the slope of the cap ceiling. If the bottom of part 4 was levelled with those of parts 1–3, a casted cap would abut on the edge of the glass ring of the MEA. The thickness of the cap ceiling could reach several millimetres, depending on the bending radius of the somewhat stiff PTFE tubing. Part 5, the top cylinder, was affixed slightly above the tubing to fully embed the tubing in the PDMS ceiling. There was a vertical slit with dimensions of 10×2 mm on the top of part 1 to allow the passage of the PTFE tubing. Part 5 had a vertical groove to allow the escape of excess PDMS precursor mix or air when finalizing the assembly after filling the template. Depending on the resulting membrane thickness, each cap consumed 4.5 ml to 7 ml of the PDMS precursor mix.

### 2.2 Polydimethylsiloxane cap fabrication

**Figure 1.**
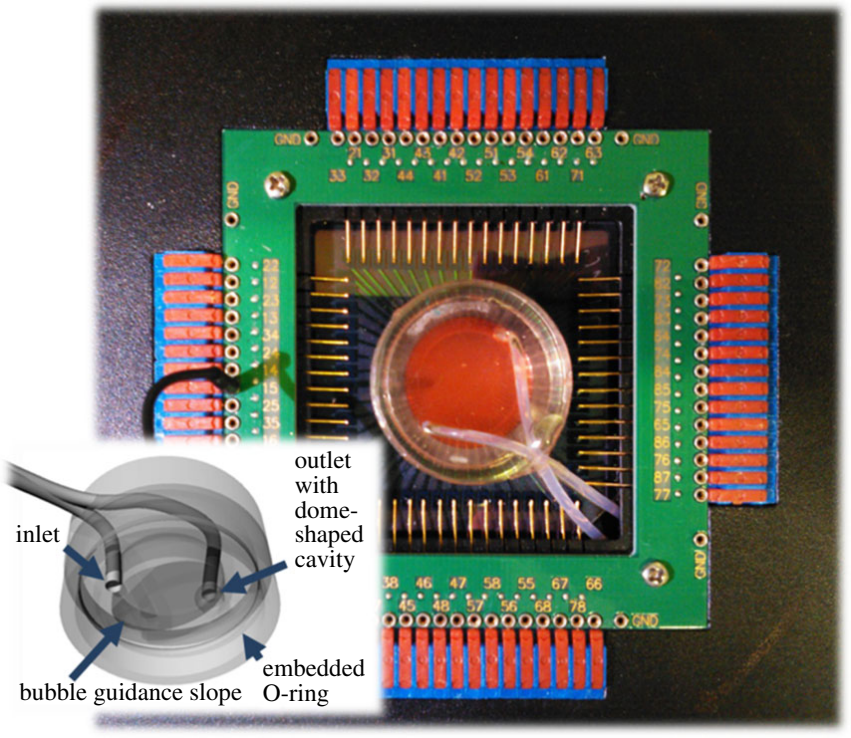
Perfusion cap features. Perfusion cap on a commercial microelectrode array (MEA) inserted into its amplifier with embedded OD 2.1 mm PTFE inlet and outlet tubes. Inset: bottom view CAD rendering of the inner cap geometry showing the central protrusion with bubble guidance slope between the inlet and outlet tube endings. A dome-shaped cavity at the outlet tube acted as a bubble trap.

PDMS properties and the cap fabrication procedure have been described in detail in a previous publication on caps without tubing [18]. We therefore summarize the main steps and only add detail for the tubing-specific differences. PDMS (Dow Corning Sylgard 184, 50 Shore A) prepolymer and the curing agent were thoroughly mixed with a metal spatula at a 10 : 1 ratio (v/v). The uniform distribution of trapped air bubbles indicated the sufficient homogeneity of the mixture, which was then degassed in a desiccator in which the vacuum was periodically broken to rupture any surfaced bubbles. PTFE tubing (OD 2.1 mm, inside diameter (ID) 1.5 mm, Supelco 20531) was reversibly clogged at one end with fishing line to prevent the entry of PDMS and was inserted into the guiding holes of part 3 (or part 4, not shown) of the moulding template (electronic supplementary material, figure S1). A small polymer half-sphere with a 2.5 mm hole was slipped onto the outlet tube to create a dome-shaped cavity in the resulting cap (figure 1, inset). To not obstruct the central optical path, both tubes were slightly bent towards the template wall and guided through the slit of part 5. The remaining slit opening was sealed with Parafilm to prevent PDMS outflow. The PDMS mixture was poured into the cavities of the assembled moulding template and cured within 24 h at room temperature, within 2 h at 60°C, or within half an hour at 85°C. After curing, the cap with its two PTFE tubes was released from the template at room temperature by pushing the slidable template components upward with the help of a rubber bottle stopper placed underneath and with 96% ethanol as a release agent. The tube endings were fitted with male Luer lock injection sites (WPI, 14034-40) with replaceable turnover flange stoppers (Carl Roth, EE00.1, Ø7.1 mm) via short pieces of Viton tubing (Cole-Parmer, 06435-01).

### 2.3 Preparation and plating of rat hippocampal cell suspensions

Where not stated otherwise, chemicals were bought from Life Technologies. Pregnant Sprague Dawley rats (CD IGS, Charles River) were anesthetized and sacrificed by cervical dislocation 18 days after conception. Following standard tissue dissociation protocols [19], their embryos (E17/E18) were harvested, put on ice in Hank's balanced salt solution (HBSS) and decapitated. After removing the meninges, the hippocampi were extracted, minced and transferred to fresh HBSS and dissociated into single cells using 0.25% (w/v) trypsin in HBSS buffer. After incubation for 10 min at 37°C, the trypsin was deactivated by 0.25 mg ml^−1^ (final concentration) soya bean trypsin inhibitor along with 0.01% (w/v) DNase (Sigma). Cell suspensions were prepared by sequential trituration (15–20 times) using three fire-polished Pasteur pipettes with decreasing diameters. Cells were then centrifuged at 200*g* for 5 min and the pellets were resuspended in Neurobasal medium (NBM) containing 2% B-27 serum-free supplement, 1 mM penicillin/streptomycin and 2 mM Glutamax. MEAs (30/200iR, Multi Channel Systems) carrying an OD 24 mm glass ring as a culture medium container were autoclaved and subsequently hydrophilized beforehand by a short O_2_ plasma treatment (0.3 mbar, 1 min, 60 W, 2.45 GHz, Diener plasma GmbH) and thereafter were coated with a 10 μl drop of a poly-d-lysine (0.1 mg ml^−1^) and laminin (5 μg ml^−1^) mix in ultrapure sterile water. Drops were allowed to dry in the vacuum of the plasma chamber. Soluble coating components were thoroughly rinsed with ultrapure sterile water. The MEA was dried again in the vacuum of the plasma chamber before plating the cells at a final density of approximately 60 000 cells mm^−2^. The cells were protected against medium evaporation by PDMS caps without perfusion functionality [18] and were allowed to settle for less than 10 min in a humidified (92–95% relative humidity (RH)) incubator at 37°C in a 5% CO_2_ in air atmosphere before the pre-warmed cell-culture medium was added.

### 2.4 Culture and perfusion medium

Cultures were plated and grown in the above-mentioned NBM. For the benchtop experiments and control cultures, the medium contained a suitable pH buffer (l-histidine, Fluka 53370; 4-(2-hydroxyethyl)-1-piperazineethanesulfonic acid (HEPES), Sigma H0887). In the first experiment, the pH of the buffered media was adjusted to a physiological pH of 7.4 at room temperature without its preconditioning to ambient CO_2_ levels (0.038%). To avoid pH drift in the second experiment, the medium was transferred to a glass beaker sealed by Parafilm and shaken for at least 4 h in a 37°C water bath to precondition the medium to ambient CO_2_ at physiological temperature. Then, HEPES was mixed in at a final concentration of 10 mM. Owing to the temperature dependency of the p*K*_a_ of most buffers [20], the pH was titrated to physiological pH at 37°C with 1 M HCl (8 μl ml^−1^) by comparing the colour of the medium (w/phenol red) with that of a control medium in a sealed flask at the same temperature. The osmolality was controlled with a vapour pressure osmometer (Vapro 5520, Wescor) before and after the pH adjustment and found to stay in a physiologically acceptable range between 220 and 260 mOsmol kg^−1^. The pH-adjusted media stocks were sterile-filtered through 0.2 μm syringe filters and stored in a standard refrigerator in airtight containers (e.g. Falcon tubes, capped syringes) for later use. The buffers were found to stabilize the pH equally well at ambient CO_2_ levels and in the control cultures that were kept in a humidified 5% CO_2_ incubator. l-histidine was used for the entire perfusion period of the first hippocampal culture, HEPES for the second.

### 2.5 Culture maintenance

Control cultures and some of the perfusion cultures were kept in a humidified (92–95% RH) incubator at 37°C in a 5% CO_2_ in air atmosphere prior to their insertion into the perfusion platform or their placement on the heating pad. Where necessary, non-buffered medium was replaced by one of the chemically buffered media before transfer. All MEAs that were stored in the incubator either carried a gas-permeable PDMS cap without tubing [18] or a perfusion cap. The caps were tightly seated around the MEA glass rings to prevent contamination and evaporation. Depending on the colour of the phenol red pH indicator, up to half of their medium (500 μl) was exchanged once a week, either by temporarily removing the caps without tubing or by manual syringe-driven perfusion through the tubing of perfusion caps.

### 2.6 Perfusion parameters

For MEAs with 7 mm high and ID 20 mm glass rings, the total cell-culture volume after cap placement varied for different inner cap geometries between 1.6 ml and 2.2 ml. Approximately, 88 μl had to be added for every 50 mm of the ID 1.5 mm PTFE tubing. Where necessary, the materials were handled and assembled on a sterile workbench to avoid contamination. For the long-term recording and imaging experiments, the tubeless PDMS cap was replaced by a PDMS perfusion cap. During cap placement and chamber filling, the Luer lock injection sites were temporarily removed from both tube ends to avoid pressure build-up and air bubble entrapment, which tended to slow down or entirely obstruct the fluid flow. Both the chamber and the tubing were filled through the inlet tube with buffered medium from a syringe before reattaching the outlet and then the inlet injection sites.

In the first perfusion experiment, a short (less than 10 mm) silver wire was inserted into the outlet septum holder to reduce the contamination risk should medium backflow from the non-sterile waste container (e.g. 15 ml Falcon tube). To prevent Ag-ion-related cytotoxicity [21], the wire was replaced with an ultraviolet A light emitting diode (UVA LED) (405 nm, 500 mcd, Conrad Electronics) in follow-up experiments [22]. This was attached by a short piece of thick tubing with a Ø 5 mm hole that was wrapped around the PTFE tubing behind the outlet septum (figure 3).

All perfusion cultures were kept at 35.5–36.5°C with an uncontrolled, but constant vertical T-gradient through the culture dish. They were either perfused automatically every eight hours (first hippocampal culture, 7 DIV+) or continuously (second hippocampal culture, 3 DIV+). With few exceptions, the exchanged volume of the above-mentioned chemically buffered media did not exceed 500 μl per day. If possible, the fluid level of the waste was kept level with the embedded end of the outlet tubing in the perfusion cap to avoid the build-up of negative pressure in the culture chamber, which could drain it in the case of any upstream leakage.

For the first experiment, the perfusion medium was stored at room temperature in a 100 ml glass bottle with a PDMS membrane (Ø 30 mm, 3 mm thick) in its cap to allow for the pH adjustment of the medium at ambient conditions without risking contamination. The flow pressure of the gravity-driven flow was adjusted by hanging the supply bottle approximately 5–20 cm above the MEA. The same type of PTFE tubing used for the perfusion caps was moulded into the bottle caps with PDMS membranes as supply lines. PTFE tubing also led from the perfusion cap outlet to the waste container (e.g. a Falcon tube). It was dipped into the waste medium to prevent clogging. Its position was slightly below the MEA surface to keep negative pressure on the outlet low.

In all cases, the PTFE tubes were interconnected with a combination of autoclavable Viton adaptor tubing (Cole-Parmer, 06435-01), polypropylene Luer fittings and syringe needles (OD 0.9 mm, G20) that pierced the cap silicone septa. This needle diameter allowed for sufficient flow while causing the least damage to the septa, thereby lowering the risk of leakage at the penetration site.

**Figure 2.**
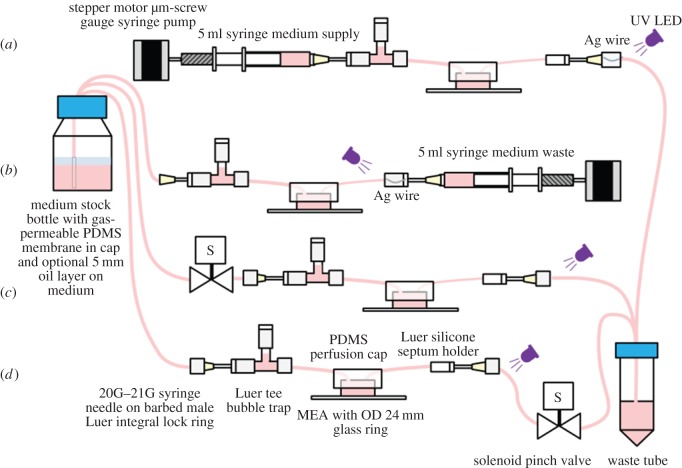
Perfusion configurations. (*a*) A microliter syringe pump, based on a stepper motor driving a micrometre screw gauge, produced an approximately constant medium exchange at very low flow rates. (*b*) Alternatively, the microliter syringe pump controlled the flow by collecting the waste, while gravity supplied the medium from a stock bottle. (*c*,*d*) Gravity-driven flow with a computer-controlled solenoid valve for timed medium exchange. The valve could be placed either between the medium supply and inlet tube (*c*) or between the outlet tube and waste container (*d*). The first arrangement prevented large spills in case the cap was not seated correctly. Depending on the height of the medium supply, the second arrangement elevated the chamber pressure thereby attenuating bubble build-up.

The first hippocampal culture was exposed to timed perfusion using a normally closed, computer-controlled (Velleman relay card K8056, Abacom Profilab) solenoid pinch valve (Model 360P071-21, NResearch Inc.). A short piece of soft silicone tubing was inserted into the PTFE tubing endings between the cap outlet and the waste. Placing the pinch valve at the outlet tubing (figure 2*d*) maintained positive pressure within the culture chamber. This helped in limiting the formation of bubbles and driving out any trapped bubbles at the risk of not being able to stop upstream medium leakage. Owing to such upstream leakage problems, the pinch valve was positioned between the medium supply and the cap inlet during the second half of the timed perfusion experiment (figure 2*c*). Volume replacement in the culture compartment was controlled by a stop-go flow. Flow rates were dictated by the opening time of the valve, the ID of the tubing and the relative height of the supply medium level with respect to the cell culture. They were empirically set to approximately 200 μl per opening of the pinch valve.

The constant perfusion experiment was based on a custom-made stepper motor-driven [23] 5 ml syringe pump. A micrometre screw gauge (MW-Import, 10-000-500-100-P) attached to a custom-made syringe holder for 0.5–5 ml syringes (inspired by World Precision Instruments, model MMP) was actuated by a stepper motor (1.8°/step). Stepping intervals were set by a timer circuit (based on National Semiconductor, LM555), which triggered the input of a stepper motor controller integrated circuit (National Semiconductor, SAA1042). Perfusion rates were set to less than or equal to 250 μl per day. An additional Luer T-piece was inserted directly behind the inlet septum holder as a bubble trap (figure 2*a*).

### 2.7 Multichannel microelectrode array electrophysiology and spike train analysis

Extracellular signals were recorded and processed by a commercial 60-channel, 1 Hz–3 kHz bandpass filter-amplifier data acquisition system (25 kHz sampling rate per channel) (Multi Channel Systems, MEA60-Up). The MEA socket in the base plate featured a resistive heating element and a Pt-100 temperature sensor. An external T-control unit (Multi Channel Systems, HC-1) kept the temperature of the socket surface at less than or equal to 36.5°C. The amplifier was mounted on a fixed, custom-made stage of an inverted microscope (Zeiss, Axiovert 200). This allowed the firm positioning of the amplifier with respect to the microscope optics to stabilize the region of interest (ROI) over time. To avoid the need to lift the cap during the MEA insertion, a custom-made Al spacer was placed between the base plate and the amplifier stage to create a gap for the free passage of the cap tubing (electronic supplementary material, figure S2). For the same reason, the lower right corner of each MEA was diagonally removed with a diamond pen. This strategy allowed the independent handling of the amplifier without lifting the cap or disconnecting the perfusion line. A grounded metal cap with central hole for illumination was placed onto the amplifier as a Faraday shield. Additionally, two grounded microclips (Conrad, 102407) were connected to both metal syringe needles at the inlet and outlet septa. A grounded wire mesh sock surrounding the tubing reduced the level of noise picked up by the fluid lines.

To reduce the data file size, only upward (positive) and downward (negative) spike cut-outs from each of the available recording electrodes were stored in 1 or 5 min packets. These consisted of 5 ms pre-spike and 5 ms post-spike fragments after first threshold crossing at ±5.5 s.d. from peak-to-peak noise. Only the downward threshold-crossings were analysed. The spike trains were transformed into time stamps using NeuroExplorer (Nex Technologies). After automatically identifying and removing simultaneous timestamps from all channels (NotSync function in NeuroExplorer) that occurred on at least four reference channels within ±10 ms owing to electrical or handling artefacts (e.g. the temporary removal of the Faraday shield), the resulting datasets were sequentially merged to generate 12 h time stamp packets for further analysis. Cumulative activity on all active channels was calculated by counting the number of time stamps within the subsequent 1 min bins. A channel was considered active if it recorded at least 30 spikes in 12 h. Most data processing steps were automated through NeuroExplorer scripts. All numbers were transferred to Microsoft Excel for plotting.

### 2.8 Imaging

To reduce spatial displacement over time, the microscope stage was replaced with an optical breadboard. After coarse alignment with the microscope optics, the amplifier was firmly screwed to the breadboard. Time-lapse pictures were captured using a remote-controlled (Breeze Systems, PSRemote) digital consumer camera (Canon, G2/G9) attached to the camera port of the microscope. Initially, the cultures were constantly illuminated by the microscope's built-in halogen lamp. As the bulbs tended to burn out after a few days, the illumination was changed to a high-intensity LED light (IKEA, Jansjö) with a supply cable that was guided through a programmable relay (Conrad Electronics, C-Control). To reduce the light-induced degradation of the chemical buffer [24,25], the light was switched on for only 30 s within a time-lapse interval of 5 (first experiment) or 3 (second experiment) min. During this period, a picture was taken. ROIs were cropped (XnView) and assembled into movies (Serif MoviePlus Starter Edition, Microsoft Movie Maker) using image durations of 0.03 s (first hippocampal culture) or 0.01 s (second hippocampal culture). Difference-images were generated with NIH's ImageJ calculator function. Fluorescence images were acquired at an inverted Leica TCS SP5 AOBS TANDEM confocal microscope and were processed with the Leica Neurolucida, NIH ImageJ and Adobe Photoshop CS3 software packages.

### 2.9 Immunofluorescence assay

Cells were fixed with 4% paraformaldehyde and 3% sucrose in phosphate buffered saline (PBS) for 10 min at room temperature (RT) and permeabilized with 0.1% Triton X-100 in PBS for 5 min at RT. Samples were blocked for 30 min in immunofluorescence (IF) buffer (3% bovine serum albumin, 2% goat serum in PBS). Primary and secondary antibodies were separately diluted in IF buffer. Samples were incubated with primary antibodies overnight, washed three times with PBS, incubated with secondary antibodies for 1 h, washed twice with PBS and rinsed with water. Samples were mounted in ProLong® Gold Antifade Mountant with 4′,6-diamidino-2-phenylindole (DAPI) (Life Technologies, P-36931). In the case of MEAs, a coverslip was placed gently on the substrate surface after adding a droplet of mounting solution on top of the cells. Primary antibodies used: polyclonal anti-neuronal class III *β*-tubulin (1 : 200, T2200, Sigma), monoclonal anti-glial fibrillary acidic protein (GFAP) IgG (1 : 400, G3893, Sigma). Fluorescent-conjugated (Alexa Fluor 488 and 647) secondary antibodies (1 : 1000) were purchased from Molecular Probes (Life Technologies).

## 3. Results

The perfusion platform is based on a transparent, gas-permeable, replica-casted PDMS cap with embedded PTFE inlet and outlet tubes (figure 1; electronic supplementary material, figure S1*b*) carrying septa injection sites at their ends. An O-ring feature ensures that caps are firmly seated on the cell-culture vessel wall, in this case a glass ring (OD 24 mm), thereby sealing the cell culture against the outside environment. Despite its stiffness, PTFE tubing was chosen for both its low cytotoxicity and low gas permeability, thereby avoiding gas bubble formation in the perfusion lines. The end of the medium inlet tube is seated close to the cell-culture substrate surface. A slightly sloped central protrusion and a dome-shaped cavity at the outlet tube assist the guidance of gas bubbles into the waste (figure 1, inset).

**Figure 3.**
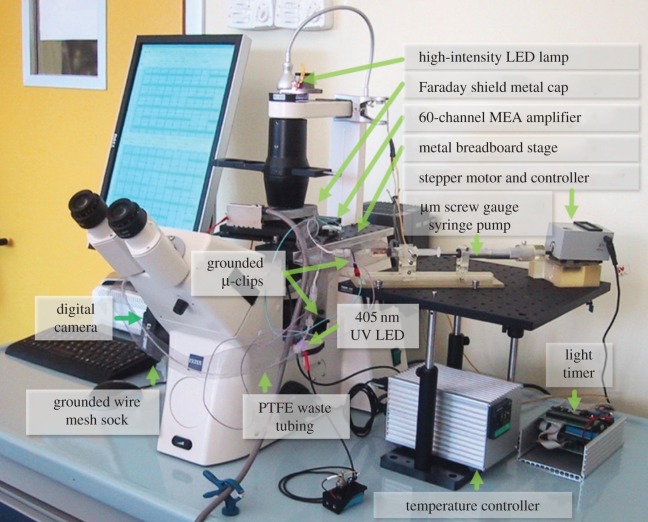
Perfusion and data acquisition platform components with their relative arrangement.

The cap is compatible with a variety of perfusion control concepts (figure 2) as compared in two experimental paradigms. The first was based on gravity-driven, timed perfusion every 8 h (figure 2*c*,*d*; electronic supplementary material, figure S3). The second relied on a constant, very slow-flow perfusion scheme based on a custom-made microliter syringe pump (figures 2*a*,*b* and 3). It was composed of a plastic syringe (1–5 ml) inserted into a three-dimensional-printed holder. The flow rates were determined by the rotational speed of a stepper motor attached to a micrometre screw pushing (or pulling) the syringe plunger (figure 2*a*,*b*).

**Figure 4.**
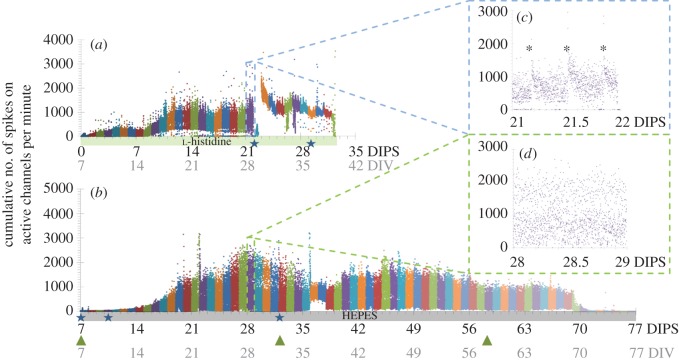
Changes in the activity over time for two different hippocampal networks in the perfusion system in ambient air. Each dot represents the cumulative activity (sum of recorded spikes) on all active channels during 1 min time increments. The bold numbers on the *x*-axis denote the days in the perfusion system (DIPS), and the grey numbers represent the overall age of the culture as days *in vitro* (DIV). Buffer types are colour-coded along the *x*-axis. Stars denote manual perfusion (of varying duration) and green arrowheads mark the syringe exchanges. Some of the high-frequency peaks probably result from undocumented threshold-crossings of temporarily noisy recording channels. (*a*) A hippocampal culture was transferred at 7 DIV from the incubator onto the perfusion platform and exposed to timed perfusion (three times per day). (*b*) The activity of a continuously perfused hippocampal culture, which had resided in the perfusion system right after cell plating, evolved similar to that of the first hippocampal culture exposed to timed perfusion; however, the number of spikes increased more slowly over the first seven days. (*c*,*d*) Representative intra-day activity fluctuations of the two cultures at the same overall age (28 DIV), but at different DIPS.

In a total of 15 perfusion experiments, four lasted less than 11 days, eight between 11 and 40 days, and three between 41 and 77 days. In the following two exemplarily reported proof-of-concept experiments, slow (less than or equal to 250 μl per day) and timed (three times per day, less than 200 μl) perfusion were compared. In the first, timed-perfusion experiment, an already differentiated hippocampal network was transferred at 7 days *in vitro* (7 DIV) from the incubator to the amplifier after addition of 10 mM l-histidine as a pH buffer. Neural activity was recorded over a period of 32 days from 7 DIV (0 days in the perfusion system=0 DIPS) until day 39 (39 DIV, 32 DIPS) (figure 4*a*). It went non-monotonously through different maturation phases. Particular events shifted the activity temporarily or permanently into different states. Network morphology was observed in 5 min time-lapse intervals from day 15 *in vitro* (15 DIV, 8 DIPS) until the end (figure 6*a*; electronic supplementary material, Movies M1 and M2). Fresh, l-histidine-buffered, but not preconditioned medium (less than or equal to 200 μl) was allowed to enter the cap every 8 h. This produced a clear and reproducible signature in the network activity (figure 4*c*, asterisks). Within 1 min, the activity dropped temporarily to zero before recovering after about 20 min.

It first exceeded and then returned to the original basal levels within 2 h. Flushing the entire chamber volume with fresh medium at 22 DIPS (29 DIV) led to a drastic activity reduction that lasted for almost 12 h (see the first star-shaped marker in figure 4*a*). On day 23, a power blackout caused the temperature to drop from 36.5°C to room temperature. After system recovery, the activity had changed to a higher rate, which lacked most of the low-frequency components found in the earlier days. Over the entire recording period, the activity was found on up to 58 of the 58 available channels, with notable intra- (figure 4*a*,*c*) and inter-day fluctuations (electronic supplementary material, figure S4, blue trace). The study was terminated at 32 DIPS (39 DIV).

In a follow-up experiment, the CO_2_-dependent medium had been first preconditioned to ambient CO_2_ levels and then been titrated to a physiological pH at physiological temperature to prevent its slow drift to basic pH in the perfusion chamber. This procedure allowed the tracking of the morphology of a second hippocampal culture immediately after cell plating. Over its 77 days lifespan in the perfusion platform, a maximum of 50 of 50 available electrodes recorded activity from 7 DIV (7 DIPS) onwards (figure 4*b*,*d*; electronic supplementary material figure S4, red trace). It evolved similarly to the activity of the first culture with a delay of about 7 days. Operations at the perfusion system such as syringe exchanges did not leave any handling signature (figure 4*b*, green arrows). In the absence of external stimuli that would alter the local microenvironment, the activity dynamics in the constantly perfused hippocampal culture spread continuously over the entire frequency range (figure 4*d*). On average, 44 channels captured activity. The experiment was terminated at 77 DIPS after a steep activity decay 7 days before (70 DIPS). Cell motility decreased around the same time (electronic supplementary material, Movies M3 and M4). A representative activity screenshot at 28 DIPS is depicted in the electronic supplementary material, figure S5.

**Figure 5.**
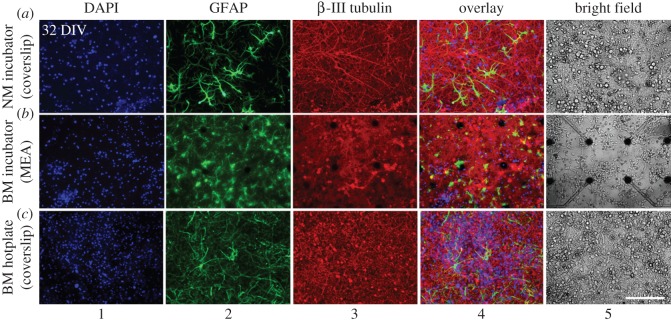
Network architectures of control cultures on glass coverslips (*a*,*c*) and MEAs (*b*) after 32 DIV. The network composition of cultures that were kept in a standard CO_2_ incubator, either with non-buffered (NM, *a*) or buffered (BM, *b*) medium, was similar to that of capped cultures with buffered medium kept on a hotplate at 37° and ambient CO_2_ level (*c*). Nuclei were stained with DAPI, glial cells with glial fibrillary acidic protein (GFAP) and neurons with *β*-III-tubulin. Right column (5): bright field images of a different set of control cultures of the same age. Electronic supplementary material, figure S6 depicts cultures at younger age. NM, normal medium; BM, buffered medium; scale bar, 200 μm.

In both cultures, network activity evolution was characterized by an overall increase during the network maturation with day-to-day variations (electronic supplementary material, figure S4) that featured local activity sinks (figure 4*a*,*b*) and intra-day state changes even under almost constant environmental conditions (figure 4*c*,*d*). These overall tendencies have been reported before [26–29]. By contrast, the activity was changed irreversibly through accidental events (e.g. a temporary system shutdown, hardware defects, drastic changes of the chemical environment after delayed nutrient exchange) (figure 4*a*). In adjunct experiments, we empirically found that an overall medium exchange volume between 200 μl per day and 250 μl per day (approx. 1/8th to 1/11th of the total chamber volume) was tolerated best. In adjunct experiments, we observed that the cultures irreversibly suffered if daily perfusion rates repeatedly exceeded the overall cell-culture volume (data not shown). Overall network composition (number of cells, glia-to-neuron ratio) of cultures in the perfusion system or on a hotplate had a similar appearance to control cultures that were kept in a standard CO_2_ incubator (figure 5; electronic supplementary material, figure S6).

**Figure 6.**
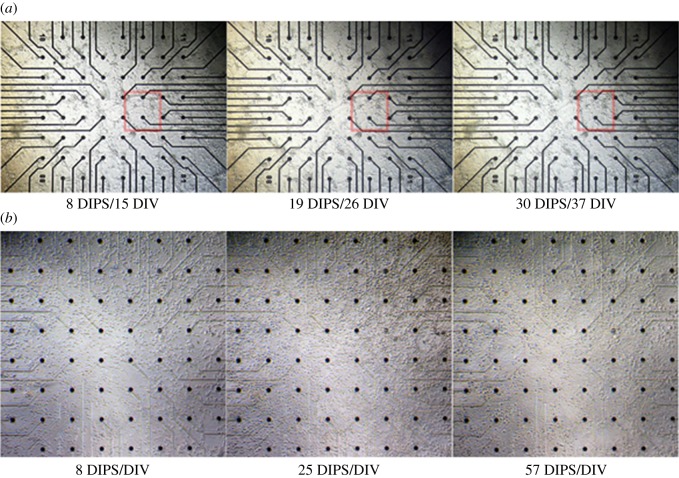
Comparative image collage of macroscopic network morphology in the two hippocampal cultures at different days. (*a*) Network morphology of the first hippocampal culture after 8 (15 DIV), 19 (26 DIV) and 30 (37 DIV) days (of a total of 32 days) in the perfusion set-up (DIPS). The clustered network arrangement is typically explained by a non-homogeneous substrate coating with adhesion mediators. While slight interconnectivity rearrangements can be found at early DIVs (red square), the overall network architecture stabilized after about two weeks. (*b*) More homogeneous network morphology of the second hippocampal culture at 8, 25 and 57 DIPS (=DIV). At its early stage, the network was still assembling, with only a few glial cells present. In this case, the macroscopic network morphology changed even at rather mature stages. Electrode diameters: 30 μm; electrode spacing: 200 μm.

In the early days, particularly during network assembly, interconnectivity rearrangements were visible (figure 6, first two columns). The macroscopic network architecture stabilized after approximately 10 DIV (figure 6*a*, second and third columns; electronic supplementary material, Movies M1 and M3).

**Figure 7.**
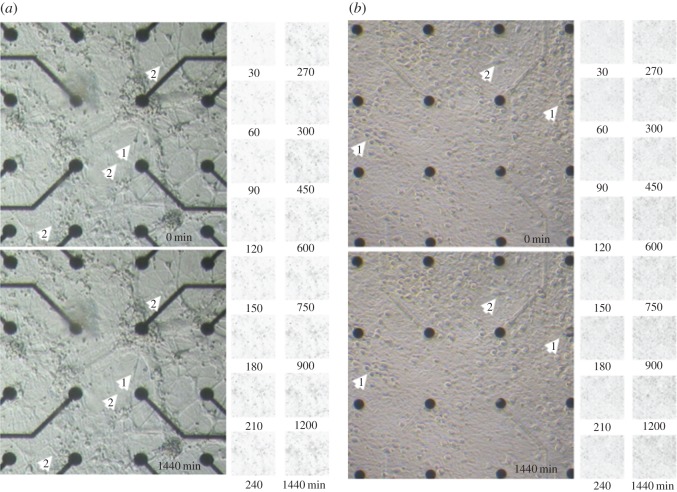
Comparative image collage of local morphological fluctuations over 24 h for the two hippocampal cultures. (*a*) Centre of the first hippocampal culture at 18 DIPS (25 DIV). (*b*) Centre of second hippocampal culture at 56 DIPS (56 DIV). The lower pictures were taken 24 h after the upper pictures at the specified DIPS. White arrowheads highlight changes visible to the naked eye. Apart from the obvious relocation of small circular-shaped motile cells (not indicated), the cells either changed their relative position (1) or the shape of their soma (2). Changes occurred locally and fluctuated around individual ‘centres of gravity’. The matrices to the right depict the differences (in black) between the first image and those taken at the indicated times in minutes. The small motile cells that travel along neural processes can be observed as slightly larger black spots in the difference maps. Over a 24 h period, increasingly darkening areas indicate overall structural changes in the network architecture. These changes are clearly visible in the electronic supplementary material, Movies M2 and M4. Electrode diameters: 30 μm; electrode spacing: 200 μm.

This observation is in contrast with the notable changes and fluctuations in the overall network activity (figure 4) and local morphological alterations (figure 7; electronic supplementary material, Movies M2 and M4). The position of the neural somata and connections fluctuated locally on the order of a few micrometres and on a timescale of minutes. These relocation and shape-remodelling events around a fixed position were more transient than permanent as the difference maps in figure 7 suggest. Motile cells, most likely of glial origin, moved in cellular contact along the neural processes, rarely passing across the areas of the cell-free substrate (electronic supplementary material, Movies M1–M4). They tended to slip occasionally underneath the neurons, thereby altering the local morphology and position of these neurons. Their sizes, non-stochastic movement pattern and non-detectable proliferation exclude bacterial origin.

## 4. Discussion

The goal of this feasibility study was the development of an inexpensive but versatile, incubator-independent benchtop perfusion system to automate cell-culture tasks and simultaneously expand the range of possibilities for the continuous and simultaneous multimodal long-term acquisition of optical and electrophysiological data. There are only few reports on optically accessible, CO_2_-independent perfusion chambers [23,30,31]. They were all designed for a particular carrier geometry or application context. We therefore conceived a simple, low-cost perfusion cap concept that can be easily adapted to the user's preferred cell-culture containers. This strategy avoids the need for modifying a set-up or existing experimental protocols and allows for the physical access to the cells by simply removing the cap.

We demonstrated that the system supported neural network viability for up to 77 days on a microscope data acquisition platform at ambient conditions without the need for any other incubation scheme. The only required parameters for neural survival and activity were a physiological temperature and pH and a sufficiently low average daily perfusion rate. In two independent system-performance evaluation experiments, neural network formation, activity onset and evolution were continuously traced using correlated time-lapse imaging and MEA electrophysiology, thereby giving a more holistic and uninterrupted view on network formation, maturation and electrophysiology not reported before.

With most of the body supply and support mechanisms missing, one of the major challenges of cell culture is the mimicry and dynamic stabilization of a complex biological microenvironment. Apart from exogenous environmental factors, the perfusion parameters determine the spatio-temporal distribution of biochemical stimuli [32] or endogenously produced signalling factors and metabolites [33], thereby steering cell fate [34]. Off-the-shelf perfusion systems usually feature high flow rates that prevent the creation and maintenance of quasi-stationary biochemical equilibria of the signalling factors in small volumes [9]. The impact of diverse pumping schemes on cell physiology has therefore received recent attention, particularly for very low perfusion rates of tens of microliters per day [35]. There is no clear agreement on which perfusion rate is most appropriate and ‘natural’ for cultured neurons residing in microfluidic devices. While slow perfusion leads to faster nutrient depletion and accumulation of metabolites [36,37], a dynamic equilibrium of local signalling factors for intercellular communication may be stabilized and shear stress may be minimized [38]. The other extreme, but common practice in cell-culture technology is to perform a partial medium exchange once to three times a week. Recent studies indicate that timed medium exchange causes the least damage to cultures [34]. We tested both paradigms. We compared stop-go flow perfusion (three times per day less than or equal to 200 μl) with a constant slow-flow (less than or equal to 250 μl per day) paradigm on neural activity progression. We found homogeneous dynamics in a constantly perfused hippocampal culture, and its activity was spread continuously over the entire activity range. By contrast, timed perfusion left clear signatures in the cumulative activity. Small instantaneous perfusion volumes (less than or equal to 200 μl) modulated the activity temporarily (figure 4*a*), while larger perfusion volumes had lasting effects (figure 4*a* at 22 DIPS). However, we did not observe an obvious difference in the day-to-day activity evolution between the two perfusion paradigms. In both cultures, the network activity followed similar subsequent developmental activity stages.

Recent reports show dramatic activity and neural response fluctuations on the timescale of seconds to days [26–29]. Signal instability is a known issue and challenge in neuroprosthetics [39,40]. We observed that the macroscopic network topography in more mature cultures was approximately stable. This suggests that activity fluctuations are not necessarily reflected by a macroscopic restructuring of the network (for its spatial freedom *in vitro* compared with intact brains). Instead, apart from activity-driven physiological events at subcellular and synaptic level [41,42], they may have their origin in the previously undocumented relative positional fluctuations of neurons and their processes with respect to the recording electrodes (electronic supplementary material, Movies M2 and M4 after 24 DIPS). In addition to the restructuring of the cytoskeleton or motile cells in close vicinity, any change in the environmental conditions and chemical composition of the microenvironment may induce transient or permanent morphological changes. If this phenomenon of relative cellular rearrangement combined with the passing of microglia were found *in vivo*, it would provide another puzzle piece in our understanding of why the quality or shapes of extracellularly recorded signals from chronically implanted electrodes fluctuate over time [39,40,43–46].

## 5. Conclusion

In this feasibility study, we presented a generalizable *in vitro*perfusion concept that was exemplarily tailored to MEA-based long-term electrophysiology and time-lapse morphology studies of network dynamics in neural cultures. This enabling technology carries potential to extend the range of experimental possibilities for most *in vitro* studies. In neuroscience, it opens the door for tracking and associating changes in network activity with changes in functional neural connectivity in real time. For instance, gradual signal shape variations may be detected more easily and be correlated to morphological rearrangement. Assessing the robustness of spatio-temporal activity patterns has implications for the computational modelling of effective connectivity and synaptic plasticity. The platform also provides a potential testbed for gaining a better and more detailed understanding on how environmental fluctuations, drugs or optical manipulation (e.g. optogenetic tools, optical tweezers or laser surgery) alter neural processing and architecture over time. Our results indicate that the outcome of an experiment (e.g. an activity modulator impact study) will very likely depend on the current basal activity level and thus on the instance in time when the experiment is performed. This poses the question of how representative results from *in vitro* experiments are, that were based on temporary snapshots of individual physiological states. Those were found to not only vary over the time course of minutes to days, but were furthermore biased by secondary handling artefacts.

## Supplementary Material

Supplementary information to ‘Incubator-independent cell culture perfusion platform for continuous long-term microelectrode array electrophysiology and time-lapse imaging’ with links to the four supplementary movies.

## Data accessibility

Original datasets are available in NeuroExplorer and Microsoft Excel file formats from Dryad: http://dx.doi.org/10.5061/dryad.n55r8.
