# Supplementary material for: Incubator-independent cell-culture perfusion platform for continuous long-term microelectrode array electrophysiology and time-lapse imaging
Source: R Soc Open Sci. 2015 Jun 17;2(6):150031. doi: 10.1098/rsos.150031 (PMC4632545; doi:10.1098/rsos.150031)
Supplement: Supplementary information to ‘Incubator-independent cell culture perfusion platform for continuous long-term microelectrode array electrophysiology and time-lapse imaging’ with links to the four supplementary movies. [file rsos150031supp1.docx]

Incubator-independent cell culture perfusion platform for continuous long-term microelectrode array electrophysiology and time-lapse imaging

Dirk Saalfrank^a,b^, Anil Krishna Konduri^a^, Shahrzad Latifi^a^, Rouhollah Habibey^a^, Asiyeh Golabchi^a^, Aurel Vasile Martiniuc^c^, Alois Knoll^c^, Sven Ingebrandt^b^, and Axel Blau^a*^

^a^ Dept. of Neuroscience and Brain Technologies (NBT), Italian Institute of Technology (IIT), Via Morego 30, 16163 Genoa, Italy, [www.iit.it](http://www.iit.it)

^b^ Dept. of Informatics and Microsystem Technology, University of Applied Sciences Kaiserslautern, Amerikastraße 1, 66482 Zweibrücken, Germany, [www.fh-kl.de](http://www.fh-kl.de)

^c^ Computer Science Dept. VI, Technical University Munich (TUM), Boltzmannstraße 3, 85748 Garching, Germany, [www.in.tum.de](http://www.in.tum.de)

*Corresponding author’s e-mail: [axel.blau@iit.it](mailto:axel.blau@iit.it)

Time-lapse movies of overall network architectures and their local spatio-temporal fluctuations

Supplementary Movie [M1](http://youtu.be/BgMX9RFqces): Time-lapse documentation of an already differentiated hippocampal network on a MEA from day 15 *in vitro* (15 DIV, 8 days in the perfusion system (DIPS)) to day 32 in the perfusion system (39 DIV, 32 DIPS) reveals a rather static overall network architecture. Images were taken every 5 minutes. In the movie, a duration of 0.03 s (1x) was assigned to each image. Microelectrode diameter: 30 µm; electrode pitch: 200 µm.

Supplementary Movie [M2](http://youtu.be/2B_b4BMDD2w): Crop of Supplementary Movie M1: Time-lapse documentation of the central region of the hippocampal network on a MEA from day 15 *in vitro* (15 DIV, 8 DIPS) to day 32 in the perfusion system (39 DIV, 32 DIPS) reveals local relocation and shape-remodeling events around a fixed position. Images were taken every 5 minutes. In the movie, a duration of 0.03 s (1x) was assigned to each image. Microelectrode diameter: 30 µm; electrode pitch: 200 µm.

Supplementary Movie [M3](http://youtu.be/8WJyXJit-J4): 77 day-long time-lapse documentation of network formation in the perfusion system immediately after plating hippocampal neurons onto a MEA. Substrate exploration by cells and formation of individual cell-cell connections can be clearly seen during the first week. The macroscopic network architecture is rather stable, while local relocation and shape-remodeling events are visible. Images were taken every 3 minutes. In the movie, a duration of 0.01 s (1x) was assigned to each image. The movie has been accelerated by a factor of 16 from day three onward. Technical problems caused an imaging gap between 16 and 24 DIPS. Microelectrode diameter: 30 µm; electrode pitch: 200 µm.

Supplementary Movie [M4](http://youtu.be/4BLOTMpniDk): Crop of Supplementary Movie M3: Zoom onto the central region of the hippocampal culture on the MEA during its 77 days in the perfusion system. Details on substrate exploration by cells and network formation are discernible during the first week. The macroscopic network architecture in the mature culture is rather stable, while local relocation and shape-remodeling events occur constantly. Images were taken every 3 minutes. In the movie, a duration of 0.01 s (1x) was assigned to each image. The movie has been accelerated by a factor of 16 from day three onward. Technical problems caused an imaging gap between 16 and 24 DIPS. Microelectrode diameter: 30 µm; electrode pitch: 200 µm.

Parts and assembly of cap replica molding template; cap properties and functionalization

Although the polydimethylsiloxane (PDMS) cap was tailored to fit the standardized cell culture glass rings (OD 24 mm) of commercial MEAs, its geometry can be easily adapted to other cell culture containers and operation conditions by additive manufacturing of custom-made CAD molding templates. The presented replica casting strategy also permits further cap functionalization by embedding electrical components, such as SMD temperature sensors, counter-, reference- or pH-wire (*e.g.*, IrOx) electrodes, heating resistors and optical components such as lenses, LEDs, color filters or pigments. Such functionalization has been described in a previous study [**1**]. Furthermore, any moldable, non-cytotoxic material with different gas and water vapor permeabilities can be used for cap construction [**2-4**]. This allows experimenters to adjust the oxygen tension to physiological levels (1-6%) [**5**]. It also eliminates PDMS-associated problems, such as the absorption of small organic molecules from the medium or leakage of uncured oligomers into the medium, as these can alter cellular physiology [**6, 7**]. The caps withstand most chemicals (including bleach) and high temperatures (tested up to 150 °C). All parts including silicone septa and polypropylene (PP) or polycarbonate (PC) Luer connectors can be sterilized using an autoclave or 70% ethanol and can be reused.

Cap molding template assembly

| 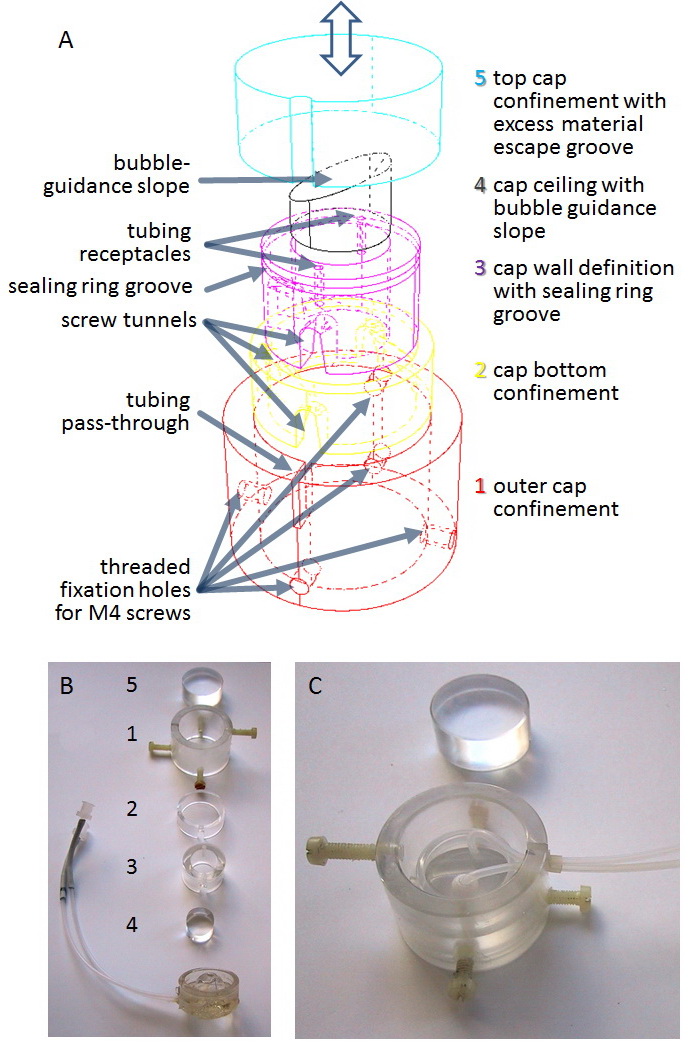 |
| --- |
| Supplementary Figure 1. Cap molding template assembly. **A.** CAD sketch of the molding template. The relative position of the five vertically slidable parts defines the vertical perfusion cap features. **B.** View of the five partially polished PMMA parts and the casted perfusion cap (bottom). **C.** Assembled template with the inserted PTFE tubing ready to be filled with PDMS. |

Elevated amplifier stage

The MEA was either kept on a temperature-controlled heating pad (not shown) or mounted on a commercial amplifier with an integrated heating stage in its base (MEA60-Up, Multi Channel Systems). To avoid the need for removing the cap from the MEA during its insertion into the amplifier, the top plate of the amplifier was lifted a few millimeters to let the cap tubing pass underneath (***Figure S2***). For the same reason, at least one edge of the MEA was diagonally cut away. The MEA rested on an aluminum spacer to compensate for the uplift of the amplifier stage.

| 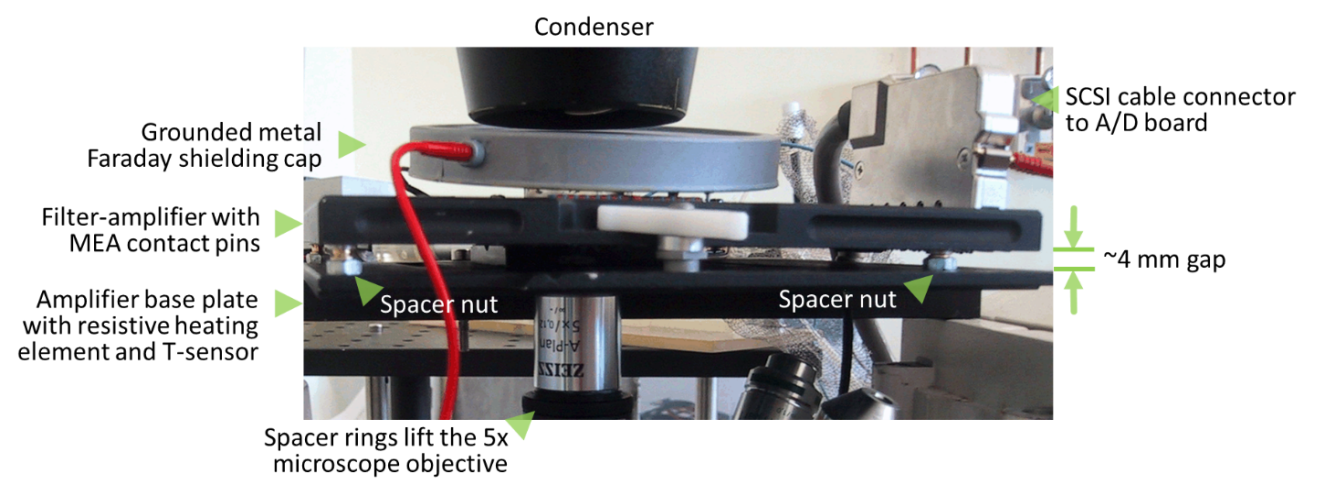 |
| --- |
| Supplementary Figure 2. The commercial amplifier stage was lifted by spacer nuts and screws to allow for the passage of the perfusion tubing. A square receptacle made from aluminium (not visible) was seated on the central heating socket of the bottom plate to lift the MEA and bring its contact pads into contact with the amplifier pins. The infinity-corrected microscopy objective (5x, Zeiss) was also raised using spacer rings (Thorlabs) to extend the focusing range of the microscope above the standard optical table height of the microscope (Axiovert 200, Zeiss). While the tubing could have been guided above the amplifier, any removal of the MEA from the amplifier would have required at least the temporary disconnection of the perfusion line or the lifting of the cap. This strategy would not only have introduced external handling artefacts, but would have also compromised the sterility if it was not performed under sterile conditions. |

Configuration for gravity-driven perfusion

| 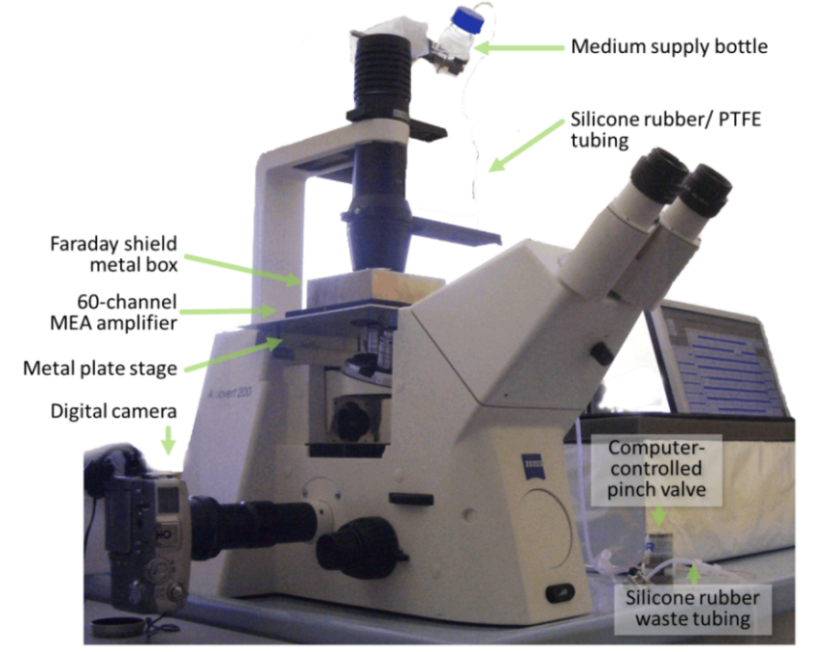 |
| --- |
| Supplementary Figure 3. Early implementation of the setup for gravity-driven perfusion experiments. It featured silicone tubing and a computer-controlled pinch valve for flow control. Due to its gas permeability, silicone tubing is prone to gas bubble formation. Wherever possible, it was replaced with PTFE tubing in the later implementations. |

Number of active sites

| 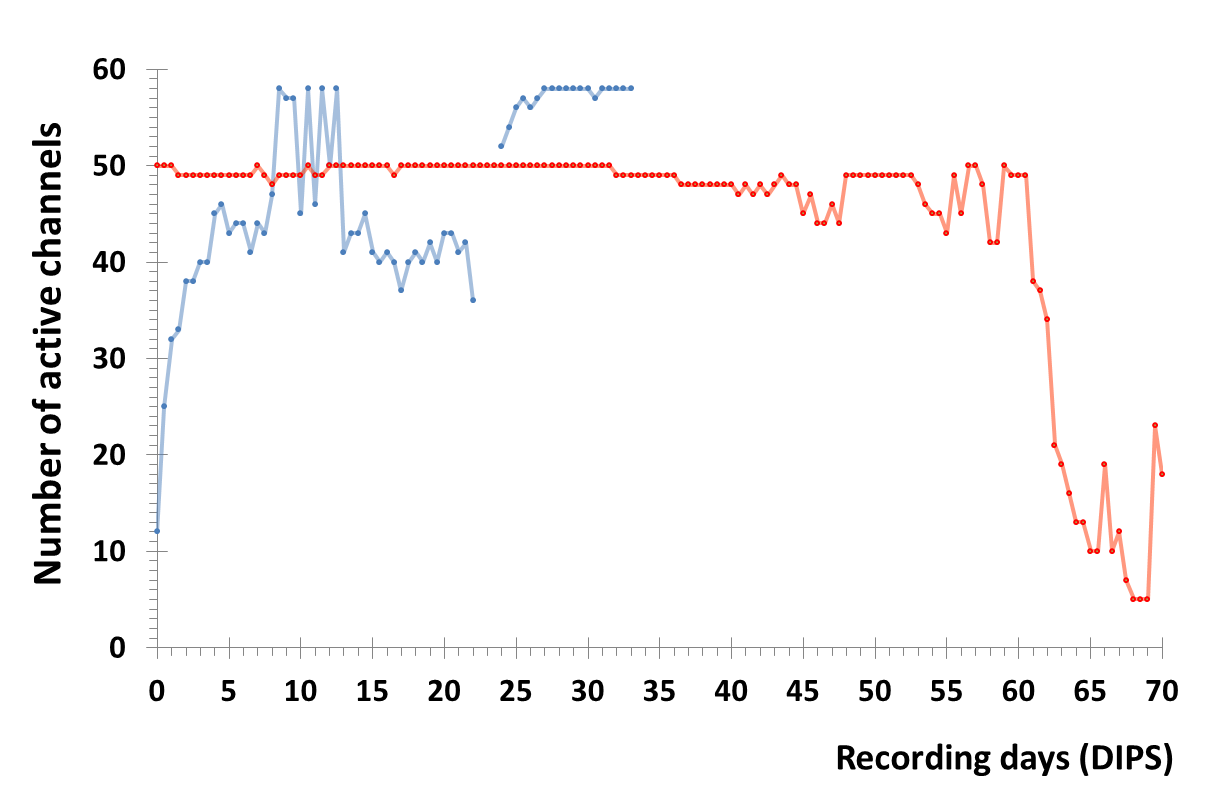 |
| --- |

Supplementary Figure 4. Number of active sites in the two hippocampal cultures over time after their insertion in the perfusion system. Each dot summarizes a 12 h period. The gap in the blue trace marks a 1.5 day power blackout. DIPS: days in perfusion system.

Activity screenshot

| 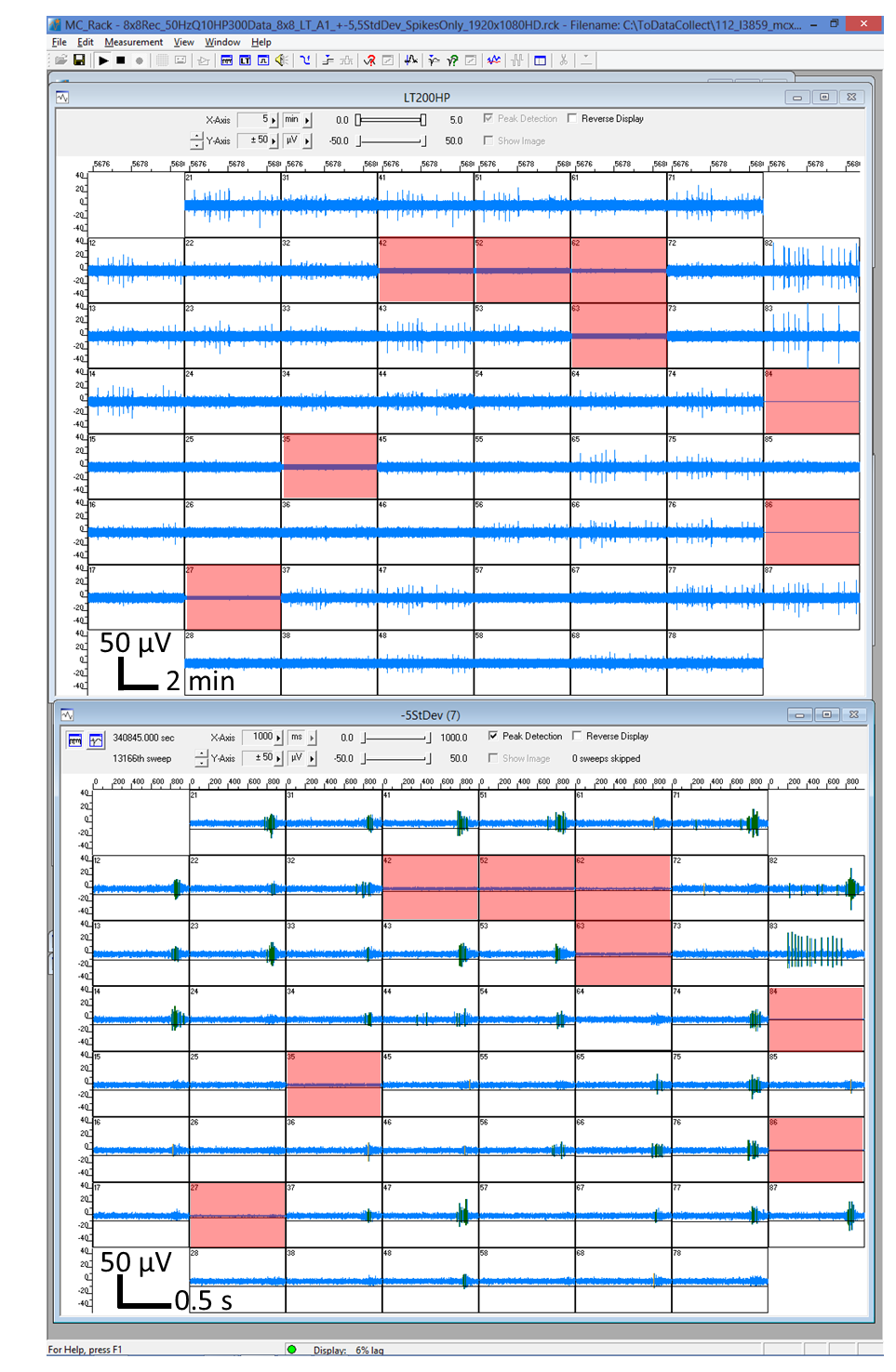 |
| --- |
| Supplementary Figure 5. Representative raw data activity recording screenshots (MC Rack, Multi Channel Systems) of the second hippocampal culture at 28 DIPS (28 DIV). Faulty and/or grounded channels (8 out of 60) are marked in red. The upper array gives a historic view of the past five minutes of activity recorded from available MEA electrodes, and the lower array gives a one-second view of the most recently recorded spike trains. Spikes and bursts crossing the ‑5.5 StDev threshold are highlighted in green. |
| Network composition of control cultures  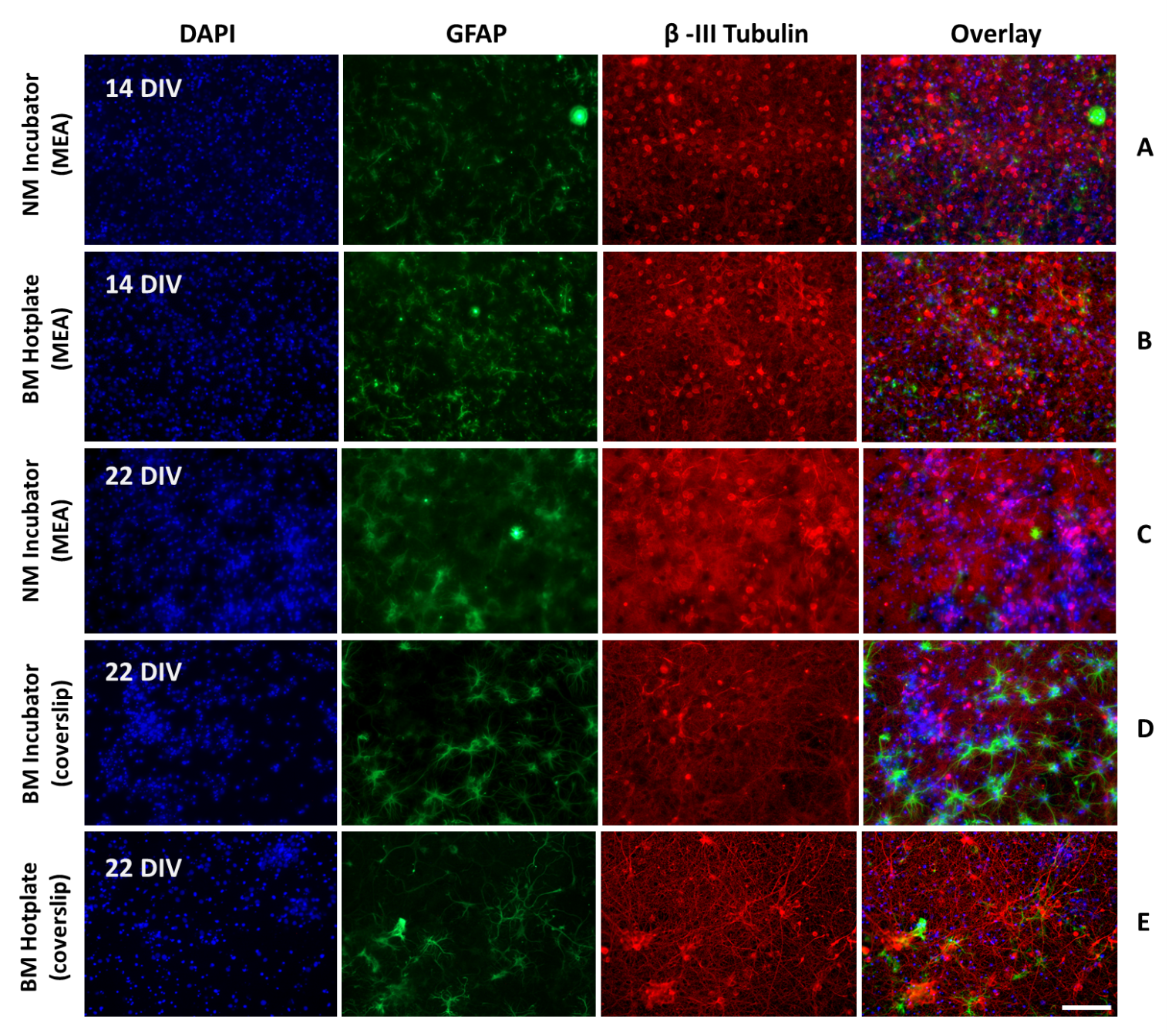 |
| Supplementary Figure 6. Network architecture of control cultures on MEAs (rows A-C) and on glass coverslips (rows D and E) after 14 DIV (rows A and B) and 22 DIV (rows C-E), respectively. Also at younger age, the network composition of cultures that were kept in a standard CO_2_ incubator, either with non-buffered (NM, rows A and C) or buffered (BM, row D) medium, was similar to that of capped cultures with buffered medium kept on a hotplate at 37° and ambient CO_2_ level (rows B and E). Nuclei were stained with DAPI, glial cells with GFAP and neurons with beta-III-tubulin. NM: normal medium; BM: buffered medium; scale bar: 200 µm. |

Perfusion system performance and typical problems

During system operation, we identified various optimization needs over time. For example, the generated gas bubbles hinder the flow [**8**]. A mismatch between the interfacial forces of the medium and the air in the PDMS and PTFE tubing often trapped bubbles in the PDMS cap, in spite of the dome-shaped outlet trap. Capillary forces favored fluid entry into the outlet tubing. The smaller the inner tubing diameter, the more this was a problem. The gas bubbles originated from different identified and tentative sources. Air may become entrapped in the septa holder during cap assembly. Air also entered through the gas- and water vapor-permeable PDMS and through the silicone septa, and may also have entered via the initially used gas-permeable silicone tubing material that connected the Luer adapters to the rigid PTFE tubing. In cases where the liquid waste level was significantly below that of the MEA, a third path of bubble entry into the chamber could have been the higher negative pressure at the cap outlet than at its inlet, which could draw air from the environment through the cap sealing ring to balance the pressure difference. Gasses may also have built up as metabolic or respiratory byproducts. Larger bubbles appeared as shadows in the images. While the chamber volume tolerates bubble accumulation without drying out the culture, a slow increase in the osmolality and an associated change in pH at the resulting ionic strength need to be taken into account. An effective compensation is the dilution of the supply medium to balance out the theoretically expected or empirically determined water loss in the chamber (Suppl. of [**1**]).

The long-term recording, imaging and gravity-driven perfusion setup went through several adjustment iterations during and between the two presented recording experiments. Those included the reconfiguration of the pumping scheme (a gravity-driven inlet and outlet with pinch valve either at the inlet (***Figure 2c***) or at the outlet (***Figure 2d***) versus a gravity-driven, unrestricted inflow combined with flow control by a syringe pump at the outlet (***Figure 2b***).

The overall platform was also prone to the temporary malfunction of some of its components. The lack of hard disk space produced interruptions in the data and image acquisition, the battery of the digital camera failed, a general power blackout occurred, automatic updates and subsequent computer restarts interfered with the results and computer memory or performance limits from too many threshold crossings stopped the activity recording program. Air bubbles trapped in too small ID tubing altered the flow properties, sometimes causing the flow to stop completely. Furthermore, medium leakage from the tubing interconnection sites or through the sealing around the cap, gas bubble accumulation in the culture compartment, pH drift, osmolality drift in the medium supply bottle due to water evaporation and condensation at the glass wall above medium level, and low-medium levels altered the environmental conditions and hence the medium composition. In consequence, datasets were partially interrupted and the recorded activity evolution could be affected by environmental fluctuations. Only the third implementation, based on a microliter syringe-pump at the inlet with Luer tee bubble trap and free medium flow from the outlet tube into a MEA-leveled waste container (***Figure 2a***), produced satisfactorily stable technical and environmental conditions over nearly the entire recording and imaging session of 70 respective 77 days.

References

1 Blau, A., Neumann, T., Ziegler, C., Benfenati, F. 2009 Replica-molded poly(dimethylsiloxane) culture vessel lids attenuate osmotic drift in long-term cell culturing. *J. Biosci.* **34**, 59-69. (10.1007/s12038-009-0009-3)

2 Massey, L. K. 2003 *Permeability Properties of Plastics and Elastomers: A Guide to Packaging and Barrier Materials*. 2nd ed: Elsevier Science.

3 McKeen, L. W. 2011 *Permeability Properties of Plastics and Elastomers*. Elsevier Science.

4 Borysiak, M. D., Bielawski, K. S., Sniadecki, N. J., Jenkel, C. F., Vogt, B. D., Posner, J. D. 2013 Simple replica micromolding of biocompatible styrenic elastomers. *Lab on a Chip*. **13**, 2773-2784. (10.1039/C3LC50426C)

5 Wright, W. E., Shay, J. W. 2006 Inexpensive low-oxygen incubators. *Nat. Protoc.* **1**, 2088-2090. (10.1038/nprot.2006.374)

6 Regehr, K. J., Domenech, M., Koepsel, J. T., Carver, K. C., Ellison-Zelski, S. J., Murphy, W. L., Schuler, L. A., Alarid, E. T., Beebe, D. J. 2009 Biological implications of polydimethylsiloxane-based microfluidic cell culture. *Lab on a Chip*. **9**, 2132-2139.

7 Berthier, E., Young, E. W., Beebe, D. 2012 Engineers are from PDMS-land, Biologists are from Polystyrenia. *Lab Chip*. **12**, 1224-1237. (10.1039/c2lc20982a)

8 Kim, L., Toh, Y. C., Voldman, J., Yu, H. 2007 A practical guide to microfluidic perfusion culture of adherent mammalian cells. *Lab Chip*. **7**, 681-694. (10.1039/b704602b)
